# Neurally adjusted ventilatory assist in pediatric intensive care units: a systematic review and meta-analysis

**DOI:** 10.3389/fped.2025.1597337

**Published:** 2025-07-10

**Authors:** Wenqian Cai, Yahui Zuo, Yan Ma, Mei Li, Meng Li, Lu Zhang

**Affiliations:** ^1^ Department of Nursing, Children's Hospital of Nanjing Medical University, Nanjing, China; ^2^ Department of Nursing, Yancheng First Hospital, Affiliated Hospital of Nanjing University Medical School, Yancheng, China; ^3^ Department of Nursing, The First People's Hospital of Yancheng, Yancheng, China; ^4^ Department of Nursing, Suzhou Municipal Hospital, The Affiliated Suzhou Hospital of Nanjing Medical University, Suzhou, China; ^5^ Emergency Department, Children's Hospital of Nanjing Medical University, Nanjing, China; ^6^ Department of Rehabilitation Medicine, Children's Hospital of Nanjing Medical University, Nanjing, China; ^7^ Department of Nursing, Nanjing BenQ Medical Center, The Affiliated BenQ Hospital of Nanjing Medical University, Nanjing, China

**Keywords:** neurally adjusted ventilatory assist, PICU, mechanical ventilation, rehabilitation, pediatric

## Abstract

**Background:**

Patient-ventilator asynchrony is a common problem in mechanical ventilation, leading to an increase in MV complications. Neurally adjusted ventilatory assist (NAVA) is a relatively new modality of mechanical ventilation that can be used for both invasive and non-invasive ventilation. There is evidence that NAVA reduces asynchronous events, but the sample size is small and the effect on specific physiological and clinical outcomes in children is controversial. Therefore, we conducted a systematic review and meta-analysis to evaluate the effect of NAVA on physiological parameters and clinical outcomes.

**Methods:**

We searched electronic databases up to 26 September 2024. Clinical trials comparing NAVA with conventional mechanical ventilation modes were included. The primary outcomes were physiological parameters, respiratory parameters, ventilator-related parameters, and other clinical outcomes. Two review authors independently extracted data and assessed study quality using the Cochrane Risk of Bias tool2. The certainty of the evidence was assessed according to the scoring methodology. Apply meta-analysis as much as possible, and use qualitative analysis when conditions are not met.

**Results:**

Eleven studies involving 224 children met the inclusion criteria for this review. Four were randomized cross-over trials, three were prospective cross-over trials, and four were retrospective studies. There were significant differences in the methods and quality of the included studies. Meta-analyses revealed significant differences in PIP, RR, pO_2_, and the asynchronous index (AI) when compared to traditional modes of mechanical ventilation. However, no significant differences were observed in FiO_2_, PEEP, TV, pH, pCO_2_, SpO_2_, EAdimax, and EAdimin.

**Conclusions:**

This systematic review and meta-analysis suggest that while NAVA has advantages for certain short-term physiological outcomes, the level of evidence remains low. Consequently, larger and higher-quality studies are necessary to identify potential short- and long-term differences between various ventilation patterns.

## 1 Introduction

Mechanical ventilation (MV) is an essential life support technique applied in the Pediatric Intensive Care Unit (PICU). The clinical application of mechanical ventilation can significantly enhance the success rate of rescuing critically ill patients while also reducing morbidity and mortality. However, in traditional ventilation modes, discrepancies between actual ventilation demands and the level of ventilation can lead to patient-ventilator asynchrony (*P*-VA). When children use pressure support mode (PS), the proportion of asynchronous time that occurs is as high as 33% (1). Among the many complications that could arise from this asynchrony are ventilator-induced lung damage and ventilator-induced diaphragm dysfunction (VIDD) (2, 3). To mitigate these undesirable outcomes and address asynchronous issues, the development and refinement of ventilation modes present a necessary challenge.

Neurally adjusted ventilatory assist (NAVA) is a relatively new mode of mechanical ventilation that can be utilized for both invasive and non-invasive ventilation. It relies on the electrical activity of the patient's diaphragm to generate respiratory effort (4, 5). Changes in diaphragmatic electrical activity at the onset of inspiration occur before changes in pressure and flow at the airway opening, allowing NAVA to have a shorter trigger delay compared to conventional ventilation modes (4). Additionally, it can adjust the intensity of ventilation to meet the patient's needs (6). This enhanced interaction between the patient and the ventilator reduces the rate of asynchrony. Previous pediatric clinical studies have demonstrated a significant improvement in synchrony with the ventilator when using NAVA (7, 8).

However, controversy persists regarding the differential effects of NAVA on physiological and clinical outcomes in children, as all studies conducted to date have small sample sizes. Therefore, this study aimed to synthesize various pediatric studies to evaluate the impact of NAVA on physiological parameters and clinical outcomes in comparison to the conventional mechanical ventilation (CMV) model.

## 2 Methods

The review protocol was registered prospectively in PROSPERO (CRD42024577790). The report of this study was presented in accordance with the guidelines of the Preferred Reporting Items for Systematic Reviews and Meta-Analyses (PRISMA), and the checklist was provided in the Supplementary Materials (9).

### 2.1 Eligibility criteria

#### 2.1.1 Inclusion and exclusion criteria

The study established the following inclusion criteria: (a) a comparative study of NAVA vs. CMV during mechanical ventilation in pediatric subjects; (b) data for at least one endpoint of interest for each group; and (c) the study must involve only pediatric patients aged under 18 years. Newborns were specifically excluded due to their classification as a distinct demographic. Furthermore, children with congenital anomalies, neuromuscular diseases, diaphragmatic paralysis, or palsy were also omitted from the analysis. We also excluded studies that inadequately reported data.

### 2.1.2 Outcomes

The outcomes of interest in this study were summarized into four broad categories: respiratory measurements, Physiological measurements, ventilator parameter correlation, and others (total duration of mechanical ventilation, length of stay in the PICU, and incidence of adverse events).

Respiratory measurements—respiratory rate (RR), fraction of inspired oxygen (FiO_2_), peak inspiratory pressures(PIP), mean airway pressure, positive end-expiratory pressure(PEEP), tidal volume (TV).

Physiological measurements—pH, pCO_2_, pO_2_, oxygen index (OI), oxygen saturation (SpO_2_).

Ventilator parameter correlation—electrical diaphragmatic activity(EAdi) including maximum EAdi (μV) and minimum EAdi (μV), asynchrony index (AI).

Asynchronous events are the lack of coordination between the respiratory activity of the patient and the mechanical assistance provided by a ventilator (10). These asynchronies are classified into five types: (a) ineffective triggering; (b) double triggering; (c) auto trigring; (d) premature cycling; and (e) late cycling (7). The Asynchrony Index (AI%) is a widely used metric for quantifying the rate of asynchrony. It is calculated by taking the ratio of the number of asynchrony events to the total number of respiratory cycles, which includes both ventilator-triggered cycles and non-triggered breaths (11).

### 2.2 Sources of information and search methodology

Until September 26, 2024, the electronic databases referenced include PubMed, Web of Science, Cochrane Library, CINAHL, CNKI, VIP, Wan Fang, and Sinomed. Depending on the database used, the search terms included MeSH terms and text words, along with free keywords combined using the Boolean operators “AND” and “OR” (Supplementary Table S1). Studies in any language and from any country would be accepted. The reference lists of the included studies and previously published systematic reviews were manually reviewed.

### 2.3 Study records

#### 2.3.1 Selection process

The database was searched by the principal investigators (CWQ). Two reviewers (CWQ, ZYH) carried out the literature screening process independently and then compared their findings based on established inclusion criteria. Disputes would be settled either by conversation or by seeking advice from a third-party examiner (MY).

#### 2.3.2 Data collection process

Using a pre-structured form, two independent reviewers (CWQ, ZYH) gathered data to collect general information and research characteristics. We performed calibration activities before the evaluation to maintain consistency among the reviewers. If needed, we reached out to the original article's author for further information. In the end, a third reviewer (MY) or a consensus method would be used to address any discrepancies.

### 2.4 Data items

The following details were extracted: study information (name of the first author, year of publication, country); type of study; sample (characteristics and number of subjects); intervention definition; control definitions; and various outcomes. If only the median and (interquartile range) ranges are reported, the normality of the data is checked using the method described by Shi et al. (12) Subsequently, the sample mean and standard deviation (SD) were estimated using the methods of Luo et al. (13) and Wan et al. (14).

### 2.5 Study risk of bias assessment

Two reviewers (CWQ, ZYH) independently evaluated the bias of randomized controlled trials (RCTs) and randomized studies using the Cochrane Risk of Bias Tool 2 (RoB 2) (15). Bias across seven domains of non-randomized intervention studies was assessed using the ROBINS-I Tool, Version 1—2016 (Risk of Bias in Non-Randomized Intervention Studies) (16). Other types of studies were evaluated using the Newcastle-Ottawa Scale(NOS). We would utilize RevMan 5.4 (Review Manager 5.4) to create a visual representation of potential bias within and between studies regarding random assignment. We did not view the lack of blinding as an issue, as blinded ventilation is virtually impossible. Furthermore, knowledge of the interventions received is unlikely to affect the outcomes selected for this review.

### 2.6 Data synthesis

Statistical software RevMan 5.4 would be utilized to combine and calculate each outcome, adhering to the statistical guidelines outlined in the current edition of the Cochrane Handbook for Systematic Reviews of Interventions. In cases where data were inadequate for meta-analysis, the results were presented in a narrative format.

#### 2.6.1 Measures of treatment effect

This study used a 95% confidence level and *p* < 0.05 as the threshold. Continuous outcomes were reported as mean differences (MDs), while dichotomous outcomes were expressed as risk ratios. When there was no discernible variation between the studies, a fixed-effect model was used; otherwise, a random-effects model was used. Subgroup analyses were conducted based on the ventilation pattern of the control group.

#### 2.6.2 Assessment of heterogeneity

We intended to use a standard Chi-square test with an alpha threshold of significance set at *p* < 0.05 to investigate heterogeneity between comparable studies. We used the *I*^2^ statistic to evaluate the degree of statistical heterogeneity, with values exceeding 50% indicating significant heterogeneity.

#### 2.6.3 Reporting bias assessment

When 10 or more studies were included in a meta-analysis, publication bias was assessed by visual inspection of the funnel plot.

### 2.7 Confidence in cumulative evidence

This study utilized the Grading of Recommendations Assessment, Development, and Evaluation (GRADE) criteria to evaluate the certainty of the evidence for each outcome (17). This framework considers the domains of bias risk, consistency, directness, precision, and reporting bias. The findings were summarized in a table of results.

## 3 Results

### 3.1 Search results and study characteristics

**Figure 1:**
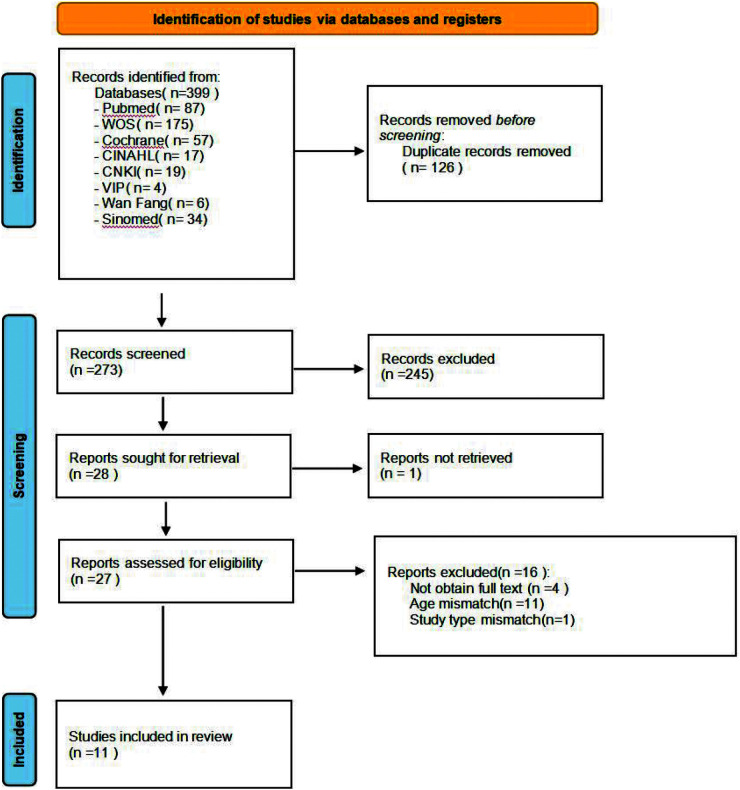
Flow diagram of the included studies.

A flowchart illustrating the studies included in this review is presented in Figure 1. Following an electronic search, 399 records were found, and after removing duplicates, 273 abstracts were assessed, of which 245 were disqualified during the title and abstract review stage for failing to meet at least one of the eligibility criteria. Among the 28 records examined, 1 could not be retrieved, and 16 were excluded (see Supplementary Table S2 for the reasons for exclusion). The two primary reasons for rejection were inconsistencies in the study population and incomplete data. Ultimately, 11 studies were included for systematic review and meta-analysis (7, 8, 18–26).

A total of 224 participants were included in this review. Three of the studies were conducted in France (22, 23, 26), three in China (18, 19, 24), three in Italy (7, 20, 21), one in Switzerland (8), and one in Canada (25). Among these eleven trials, four were randomized crossover trials, three were prospective crossovers, and four were retrospective studies. There were some differences in the inclusion criteria, as three of the studies specifically focused on comparing the effects of non-invasive NAVA in children with PICU (7, 20, 25). Table 1 presents the characteristics of each included study.

**Table 1:** Characteristics of included studies.

Study, year	Country	Study type	Paiticipants	Sample size	Treat	Control
Liet et al. 2016 (23)	France	Randomized crossover	Children after cardiac surgery	6	NAVA	CMV
Chidini et al. 2016 (7)	Italy	Randomized crossover	Children with ARF	18	NIV-NAVA	NIV-PSV
Zhu et al. 2016 (24)	China	Randomized crossover	Children after cardiac surgery	21	NAVA	PSV
Vignaux et al. 2013 (8)	Switzerland	Randomized crossover	Children in PICU	19	NAVA	PSV
Ducharme-Crevier et al. 2015 (25)	Canada	NonRandomized crossover	Children in PICU	13	NIV-NAVA	NIV
Xiao et al. 2021 (19)	China	NonRandomized crossover	Children in PICU	23	NAVA	CPAP
Spinazzola et al. 2020 (21)	Italy	NonRandomized crossover	Children with moderate ARDS	12	NAVA	PSV
Piastra et al. 2014 (26)	France	Retrospective cohorts	Children with ARDS	30(control 20, intervention 10)	NAVA	PSV
Chidini et al. 2021 (20)	Italy	Retrospective cohorts	Children with AHRF	64(control 34, intervention 30)	NIV-NAVA	NIV-PSV
Assy et al. 2019 (22)	France	Retrospective cohorts	Children who received Veno-venous ECMO	6	NAVA	CMV
Liu et al. 2022 (18)	China	Retrospective cohorts	Children in PICU	12	NAVA	SIMV

NAVA, neurally adjusted ventilatory assist; CMV, conventional mechanical ventilation; ARF, acute respiratory failure; NIV, noninvasive ventilation; PSV, pressure support ventilation; PICU, pediatric intensive care unit; ARDS, acute respiratory distress syndrome; AHRF, acute hypoxemic respiratory failure; ECMO, extracorporeal membrane oxygenation.

### 3.2 Risk of bias

**Figure 2:**
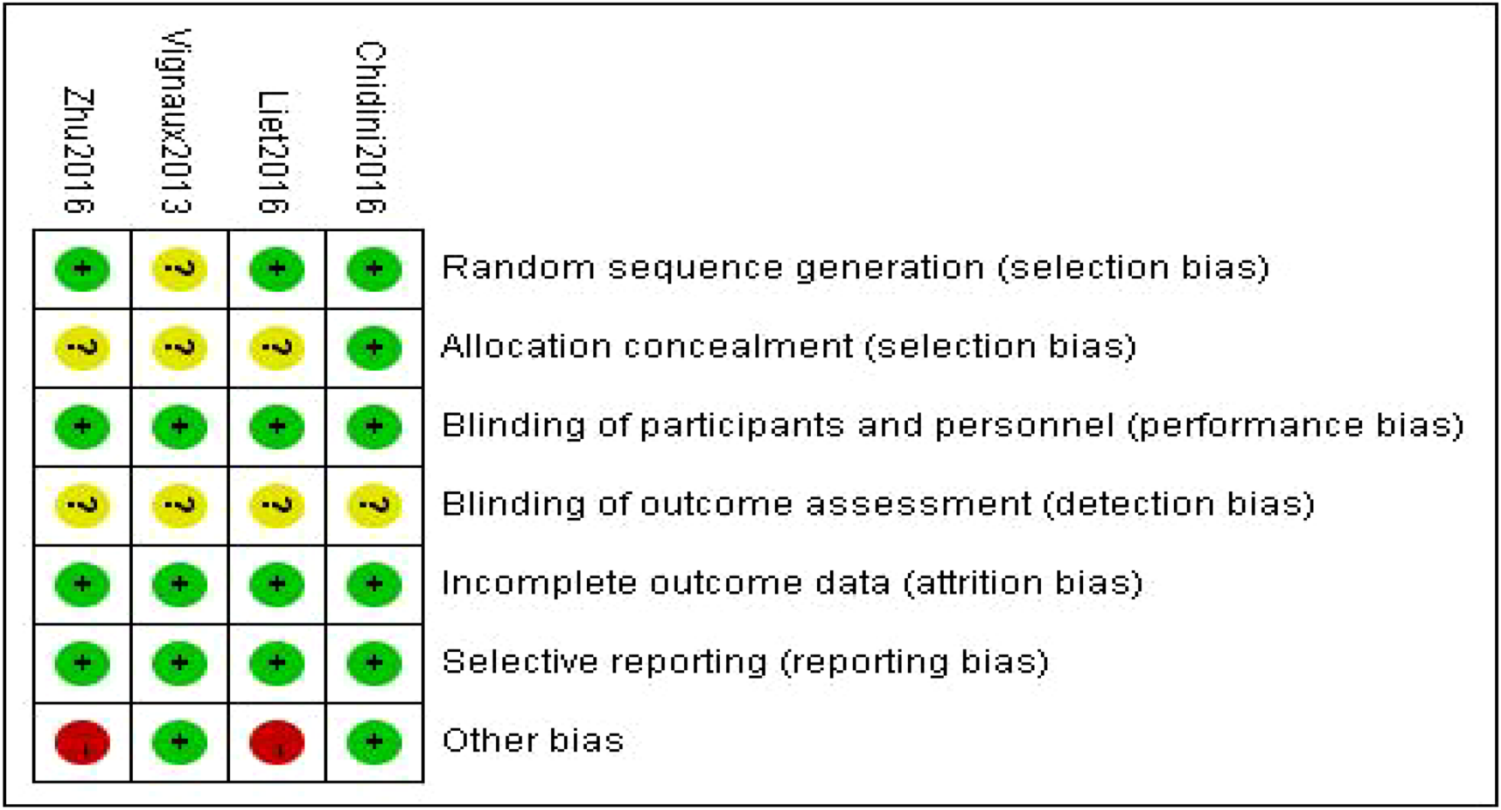
Risk of bias summary.

The RoB2 tool indicated that two of the four randomized crossover studies were at high risk of bias (Figure 2), and the source of this bias was identified as the lack of a washout period during the crossover process (23, 24). In the three non-randomized studies evaluated using the ROBINS-I tool, both exhibited a moderate risk of bias (Supplementary Table S3), the absence of a washout period during the intervention crossover phase and the failure to report planned outcomes at the conclusion of the studies further compromised study quality (19, 21). For retrospective cohort studies, the NOS scale yielded overall scores ranging from 5 to 7 points. Most studies did not include a non-exposed group for study subjects, controls for confounding factors were not clearly defined, and most studies (75%) assessed as having a moderate risk of bias in terms of adequacy of follow-up (Supplementary Table S4).

### 3.3 Quantitative analysis

Nine of the eleven studies included in the analysis participated in the meta-analysis, while one study was excluded because its data were presented in median (interquartile) form. The mean and standard deviation were not available, as the authors were not contacted to provide this information; therefore, this study was only included in a narrative format.

#### 3.3.1 Comparison 1 respiratory measurements

**Figure 3:**
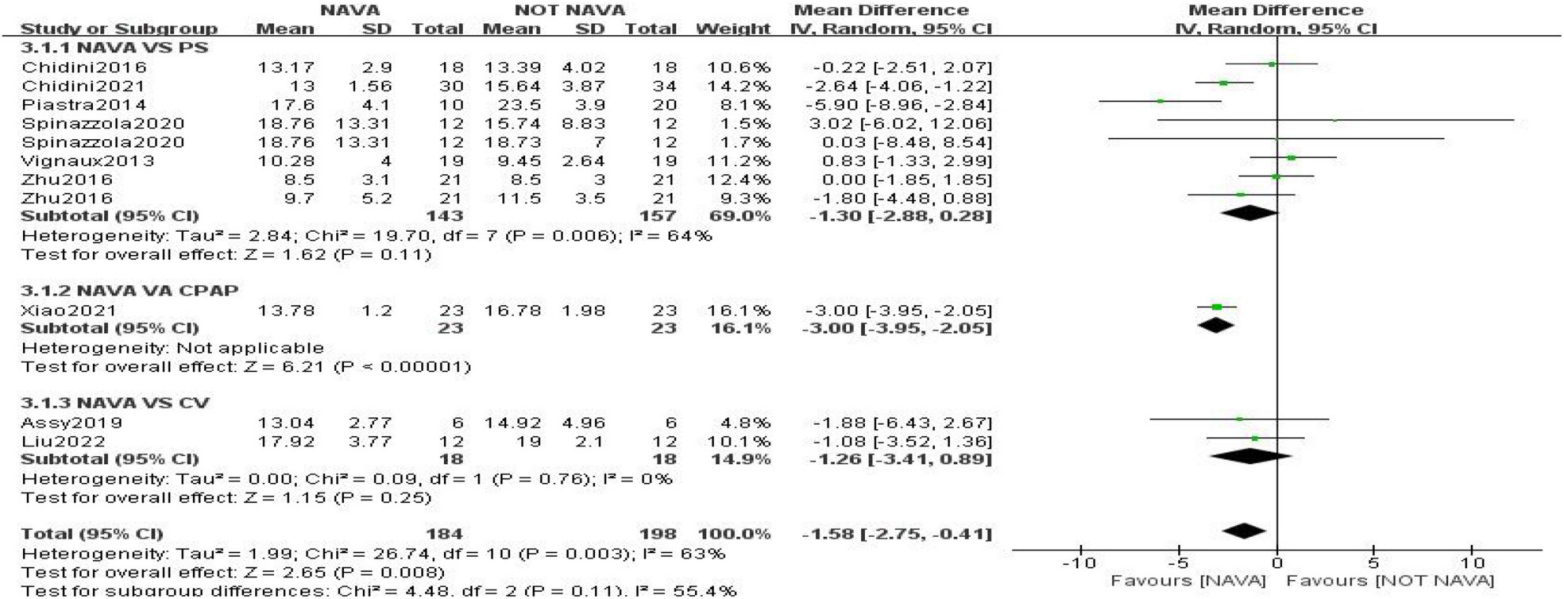
Forest plot demonstrating pooled results for PIP.

**Figure 4:**
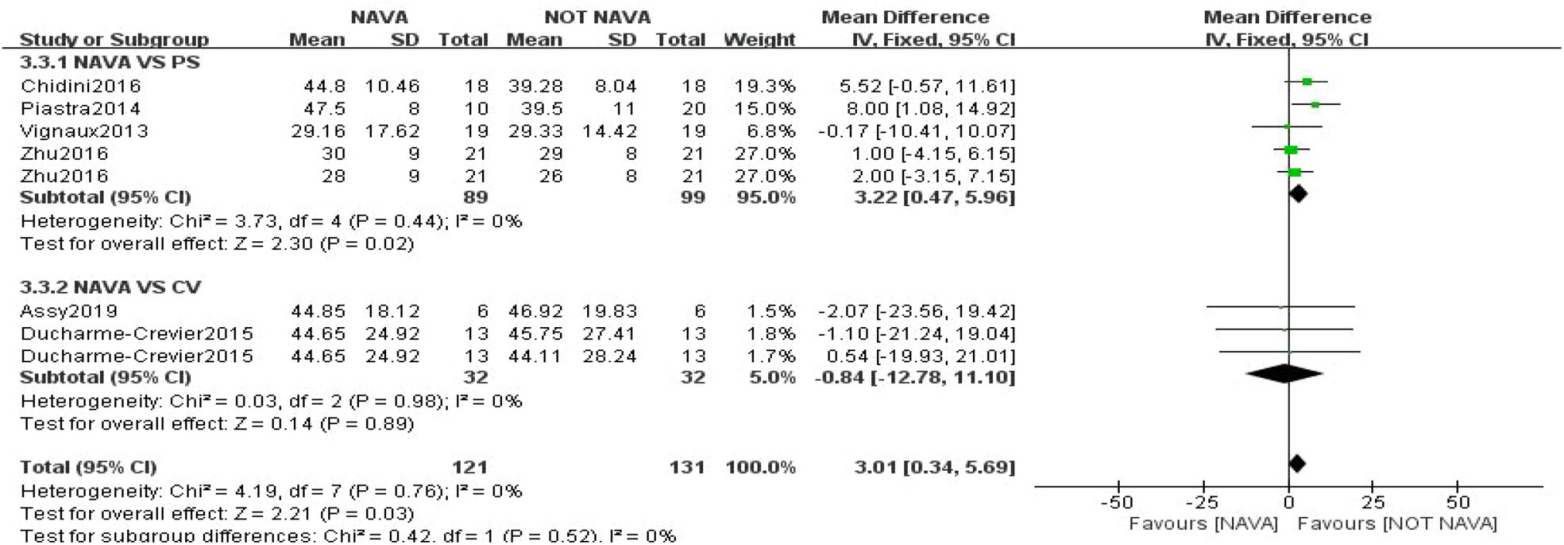
Forest plot demonstrating pooled results for RR.

The pooled analysis indicated that in nine studies involving 382 children, NAVA had a significant reduction in peak inspiratory pressure (PIP) compared to other forms of ventilation (7, 8, 18–22, 24, 26). The mean difference was −1.58 (95% CI: −2.75 to 0.41), demonstrating significance in both fixed and random-effects models, with an inconsistency index (I²) of 63% (Figure 3). Significant differences in respiratory rate (RR) were observed in NAVA (MD: 3.01, 95% CI: 0.34 to 5.69; six trials, 252 children; *I*^2^ = 0%) (Figure 4). For mean airway pressure (Pmean) (MD: −0.95, 95% CI: −1.95 to 0.05; six trials, 280 children; *I*^2^ = 76%), positive end-expiratory pressure (PEEP) (MD: 0.04, 95% CI: −0.13 to 0.21; three trials, 120 children; *I*^2^ = 20%), tidal volume (TV) (MD: 0.10, 95% CI: −0.24 to 0.44; seven trials, 288 children; *I*^2^ = 23%), and FiO_2_ (MD: −0.00, 95% CI: −0.04 to 0.03; four trials, 172 children; *I*^2^ = 55%), no significant difference was found. (Supplementary Figures S1–S4).

#### 3.3.2 Comparison 2 physiological measurements

**Figure 5:**
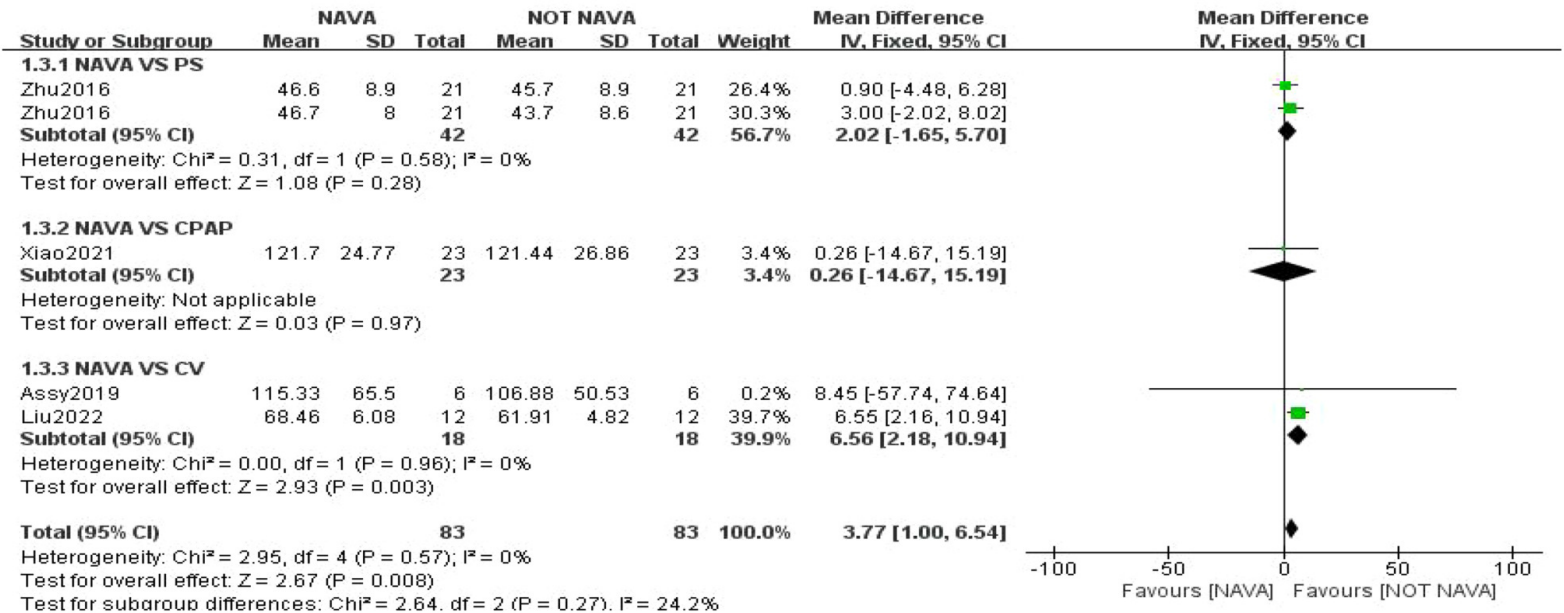
Forest plot demonstrating pooled results for pO_2_.

Physiological measurements include pH, pCO_2_, pO_2_, OI, and SpO_2_. For the results of pO_2_, our study included four studies involving a total of 166 children (18, 19, 22, 24), and the analysis showed that pO_2_ was statistically significantly higher in the NAVA group (MD: 3.77, 95% CI: 1.00–6.54). The heterogeneity test yielded an *I*^2^ value of 0%, suggesting low heterogeneity (Figure 5). For the outcome of pH, our study included four studies with a total of 174 children (21, 22, 24, 26), revealing no significant difference between the groups (MD: −0.01, 95% CI: −0.02 to 0.00; *I*^2^ = 44%). For pCO_2_ outcomes, our study included five studies involving 214 children (18, 19, 21, 22, 24), and the analysis showed no significant difference between NAVA and other modes of ventilation (MD: −0.22, 95% CI: −2.06 to 1.62; *I*^2^ = 43%). For SpO_2_ outcomes, we analyzed three studies with 126 children (22, 24, 26), which also revealed no significant difference between the groups (MD: 0.50, 95% CI: −1.12 to 2.12; *I*^2^ = 0%) (Supplementary Figures S5–S7). Regarding the results for Oxygenation Index (OI), we did not perform quantitative analyses due to inconsistencies in units across the two studies (18, 21). However, the study conducted by Spinazzola et al. (21) demonstrated a significant improvement in OI during the NAVA trial compared to PSV (*p* = 0.004).

#### 3.3.3 Comparison 3 ventilator parameter correlation

**Figure 6:**
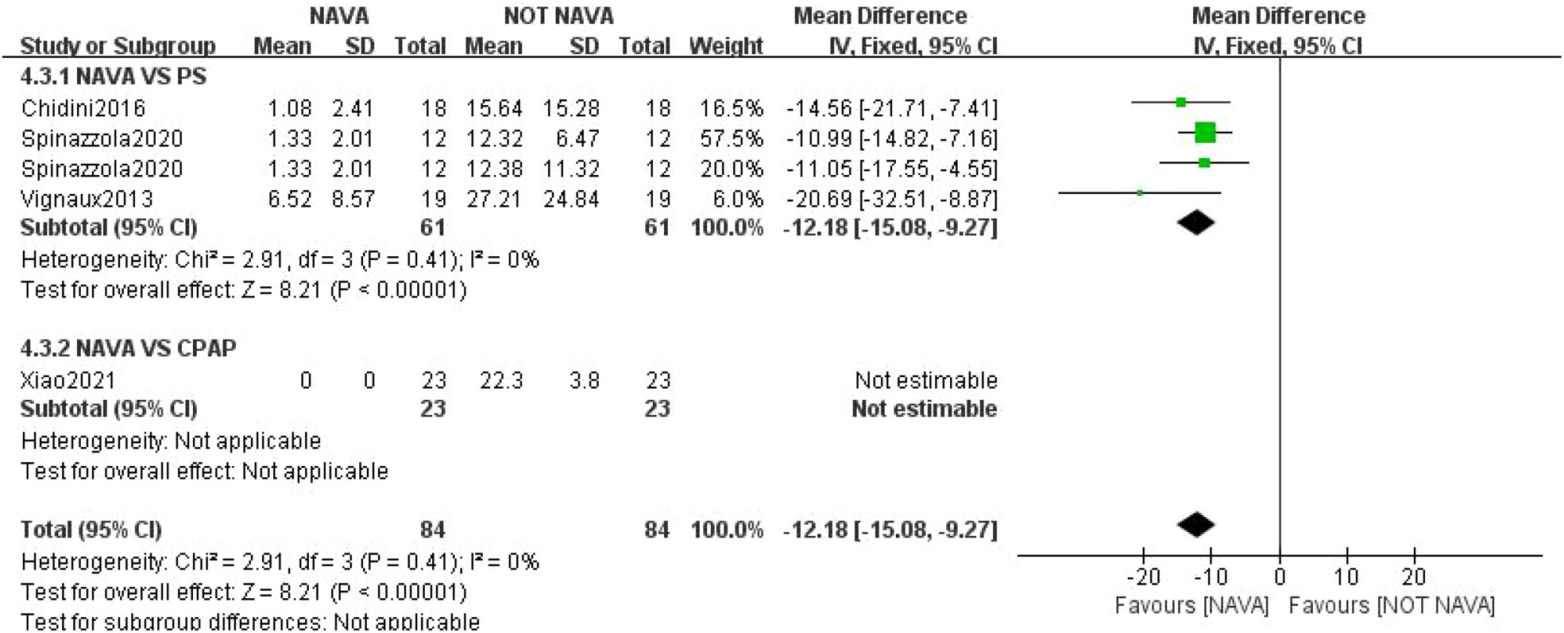
Forest plot demonstrating pooled results for AI.

For ventilator-related parameters, we primarily assessed the maximum EAdi, minimum EAdi, and the ventilator asynchronous index (AI). For AI, our study included four studies involving a total of 168 children (7, 8, 19, 21). Among these, three studies compared NAVA and PSV and only one study compared NAVA and CPAP. Compared with the PSV group, the results indicated that the NAVA group (61 participants) had significantly lower AI values compared to the PSV group (61 participants) (MD: −12.18, 95% CI: −15.08 to −9.27; *I*^2^ = 0%) (Figure 6). Studies comparing with the CPAP group also showed no asynchronous events in the NAVA group (19). Five studies reported maximum EAdi (*n* = 244) (7, 18, 21, 24, 25), revealing no significant difference in maximum EAdi between the two groups (MD: −0.04, 95% CI: −1.16 to 1.07; *I*^2^ = 0%). Three studies reported minimum EAdi (*n* = 160) (18, 24, 25), and there was no significant difference in minimum EAdi between the two groups (MD: −0.14, 95% CI: −0.36 to 0.08; *I*^2^ = 0%) (Supplementary Figures S8, S9).

#### 3.3.4 Other outcomes

Quantitative analyses regarding the duration of mechanical ventilation, length of stay in the PICU, and the incidence of adverse events during NAVA were not feasible due to insufficient data. Chidini et al. (20) demonstrated a significant reduction in PICU stay [5 [4–7] vs. 9 [6–9.4] days, *p* = 0.002] and a significant reduction in the incidence of ventilator-associated pneumonia (VAP) [5% [20] vs. 0% [0], *p* = 0.004] in the NAVA group compared to the PS group. Additionally, Liu et al. (18) reported that none of the children experienced complications related to or following the conversion to NAVA.

### 3.4 Certainty of evidence

**Figure 7:**
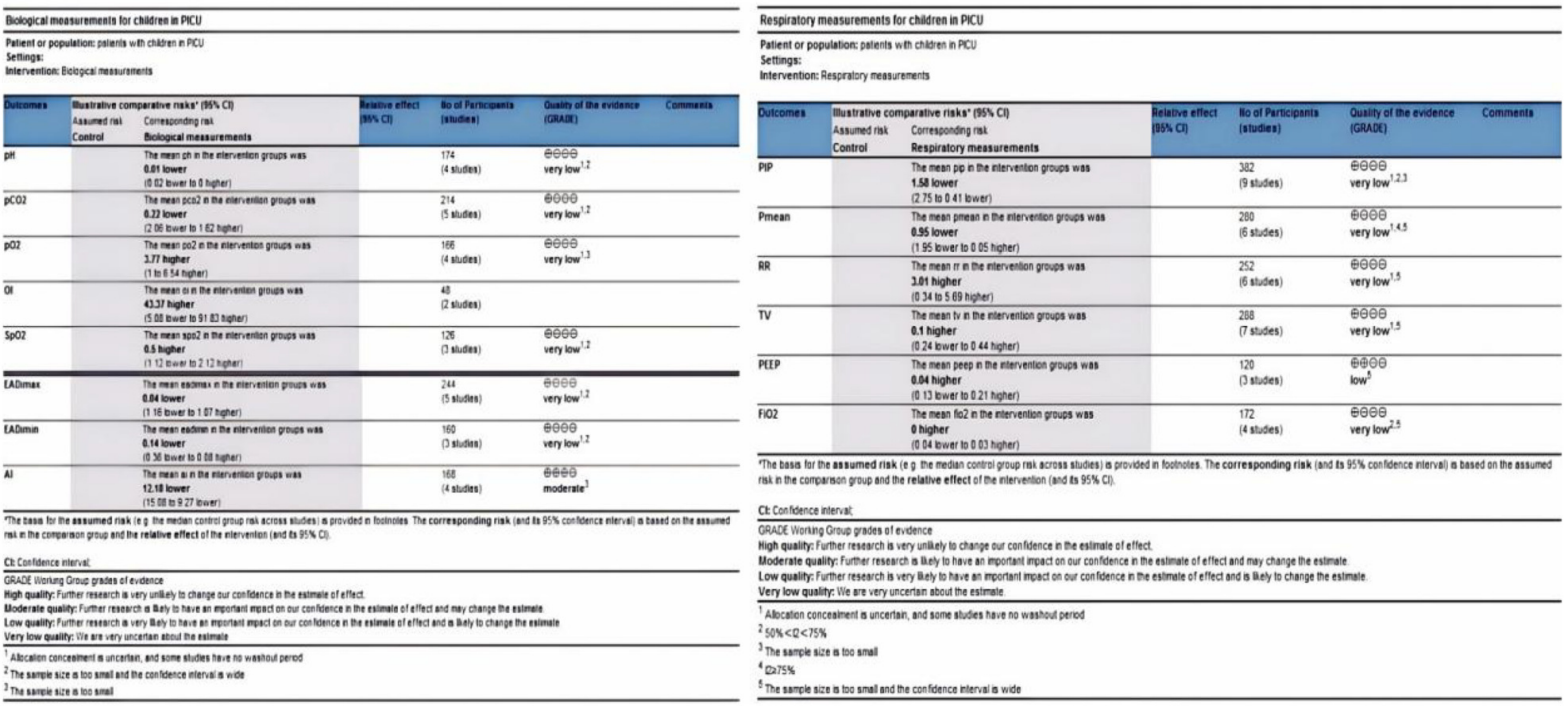
GRADE summary of findings.

The outcome summary graph (Figure 7) shows the quality of the evidence for the outcomes.

## 4 Discussion

Based on a limited number of studies exploring the effects of NAVA on children hospitalized in the PICU other than neonates, this systematic review and meta-analysis identified 11 studies involving 224 children. The results indicated significant differences in PIP, RR, pO_2_, and Asynchrony Index (AI) when compared to traditional mechanical ventilation modes. However, the overall quality of the evidence was very low, with the exception of moderate quality evidence for AI. Regarding clinically relevant outcomes, there is insufficient data to support meta-analyses demonstrating the superiority of NAVA in terms of efficacy.

Meta-analysis indicates that the use of NAVA is associated with lower peak inspiratory pressure (PIP), improved patient-ventilator synchrony, and increased pO_2_. Furthermore, we performed subgroup analyses of AI by ventilation mode, and even when PSV or CPAP was used as the comparator, NAVA produced a statistically significant decrease in AI. It improves patient comfort and reduces ventilation discomfort since it is activated by the patient's inspiratory effort and permits adaptive ventilation parameter modifications (27, 28). Consistent with our research findings, oxygenation can also be improved by improving synchronization (4). The decrease in PIP can be attributed to the simultaneous improvement in patient-ventilator interaction. NAVA reduces the work of breathing (8), leading to a decrease in PIP. If adequate gas exchange can be achieved at lower intrapulmonary pressures during MV, as is the case with NAVA, it has the potential to minimize lung damage. This limitation is also present in existing clinical studies, and we recommend conducting more extensive and long-term studies to validate the effects of NAVA on lung function. The EAdi levels are another way to evaluate the work required to breathe (29). Since children have a low threshold for diaphragm fatigue (7) and the baseline EAdi levels of the children in the study were low, indicating less excessive breathing effort, our meta-analysis did not identify a significant difference in EAdi.

In the meta-analysis, we also found that the RR of NAVA tends to be higher than that of conventional mechanical ventilation (CMV). The RR of NAVA is measured based on the EAdi signal, while the RR of conventional mechanical ventilation is determined by changes in airway flow. Animal studies have shown that under pressure support (PS), the neural RR in rabbits consistently exceeds the ventilatory RR (30). Additionally, the breathing characteristics of pediatric patients include low tidal volume, weak inspiratory effort, high respiratory rate, and short neural time (31).

There are several limitations to this study. First, our systematic review did not include any randomized controlled trials (RCTs). In terms of study design, we incorporated prospective crossover trials and retrospective analyses, both of which exhibited low study quality. This may have introduced bias into the results. Second, since more than ten studies failed to report outcomes, an assessment of publication bias was not feasible. Again, most of the included studies focused on short-term ventilation and clinical outcomes, and so far there have been no RCTs to verify the effect of patient-ventilator asynchrony on primary clinical outcomes, such as the incidence of MV-related complications, duration of mechanical ventilation, length of hospital stay in ICU or pediatric patients, except for those that have shown that NAVA improves patient-ventilator interaction and some minor physiological outcomes. Finally, while this meta-analysis highlights several advantages of NAVA, it is important to acknowledge its potential limitations. Notably, there are currently no evidence-based guidelines for NAVA settings. This absence of guidelines may indicate a lack of experience with NAVA in the included trials, which could obscure the true impact on the measurement results.

## 5 Conclusion

Overall, NAVA improved synchrony with the ventilator and improved physiological and clinical outcomes in children with MV compared to the CMV model. This study is the first comprehensive systematic review and meta-analysis to date that focuses on NAVA in critically ill children, excluding neonates. It is recommended that future research should concentrate on analyzing additional clinical outcomes and conducting larger multicenter, multisample randomized controlled trials to validate the effectiveness of NAVA.

## Data Availability

The original contributions presented in the study are included in the article/Supplementary Material, further inquiries can be directed to the corresponding authors.
