# Supplementary material for: Neurally adjusted ventilatory assist in pediatric intensive care units: a systematic review and meta-analysis
Source: Front Pediatr. 2025 Jul 10;13:1597337. doi: 10.3389/fped.2025.1597337 (PMC12286966; doi:10.3389/fped.2025.1597337)

**Supplementary Table S1: Search Strategy**

| Pubmed | #1 | (("Child"[Mesh]) OR "Pediatrics"[Mesh]) OR "Intensive Care Units, Pediatric"[Mesh] | 87 |
| --- | --- | --- | --- |
|  | #2 | ((("Child"[Mesh]) OR "Pediatrics"[Mesh]) OR "Intensive Care Units, Pediatric"[Mesh]) OR (((pediatric*[Title/Abstract]) OR (child*[Title/Abstract])) OR (PICU[Title/Abstract])) |  |
|  | #3 | ("Neurally Adjusted Ventilatory Assist)"[Title/Abstract]) OR ("NAVA"[Title/Abstract] |  |
|  | #4 | #2 AND #3 |  |
| Web of Science | (TS=("Neurally Adjusted Ventilatory Assist" OR "NAVA")) AND TS=("child*" OR "pediatric*" OR "PICU") | | 175 |
| Cochrane | "Neurally Adjusted Ventilatory Assist" OR "NAVA" in Title Abstract Keyword AND "child" OR "pediatric" OR "PICU" in Title Abstract Keyword | | 57 |
| CINAHL | #1 | SU child* OR SU pediatric* OR SU PICU | 17 |
|  | #2 | (MH "Child+") OR (MH "Pediatrics+") OR (MH "Intensive Care Units, Pediatric+") |  |
|  | #3 | #1 OR #2 |  |
|  | #4 | AB neurally adjusted ventilatory assist OR AB "NAVA" |  |
|  | #5 | #3 and #4 |  |
| CNKI | (TKA%='Neurally Adjusted Ventilatory Assist' + 'NAVA') AND (TKA%='Child' + 'Pediatrics' + 'PICU') | | 19 |
| VIP | M=('Neurally Adjusted Ventilatory Assist' OR 'NAVA') AND M=(child OR Pediatrics OR 'PICU') | | 4 |
| Wan Fang | Title or key words:("Neurally Adjusted Ventilatory Assist" or "NAVA") and Title or key words:("child" or "Pediatrics" or "PICU") | | 6 |
| Sinomed | ("Neurally Adjusted Ventilatory Assist" OR "NAVA" AND ("child" OR "Pediatrics" OR "PICU") | | 34 |

**Supplementary Table S2:** Reasons for exclusion of studies reviewed in full text

| ID | Title | Reason for exclusion |
| --- | --- | --- |
| Alander2012 | Comparison of pressure-, flow-, and NAVA-triggering in pediatric and neonatal ventilatory care | The study subjects did not match, mixed populations |
| Rosterman2018 | The impact of neurally adjusted ventilatory assist mode on respiratory severity score and energy expenditure in infants: a randomized crossover trial | The study participants did not match, neonates |
| Houtekie2015 | Feasibility Study on Neurally Adjusted Ventilatory Assist in Noninvasive Ventilation After Cardiac Surgery in Infants | The study participants did not match, neonates |
| Baudin2014 | Impact of Ventilatory Modes on the Breathing Variability in Mechanically Ventilated Infants | The study subjects did not match, mixed populations |
| Kallio2015 | Electrical activity of the diaphragm during neurally adjusted ventilatory assist in pediatric patients | The study subjects did not match, mixed populations |
| Kallio2015 | Neurally adjusted ventilatory assist (NAVA) in pediatric intensive care—A randomized controlled trial | The study subjects did not match, mixed populations |
| Clement2011 | Neurally triggered breaths reduce trigger delay and improve ventilator response times in ventilated infants with bronchiolitis | The study subjects did not match, mixed populations |
| Zhu2009 | Application of Neurally Adjusted Ventilatory Assist in infants who underwent cardiac surgery for congenital heart disease | The study subjects did not match, mixed populations |
| Zhang2017 | Application of neurally adjusted ventilatory assist in pediatric clinical practice | Study participants did not match, and age ranges were not specified |
| Riwaaj LAMSAL2021 | Non-invasive Neurally Adjusted Ventilatory Assist (NAVA) in the Pediatric ICU: Assessing optimal Edi compliance | The study subjects did not match, mixed populations |
| Cammarota2016 | New Setting of Neurally Adjusted Ventilatory Assist during Noninvasive Ventilation through a Helmet | Study subjects do not match, adults |
| Schibler2013 | A randomised controlled study investigating neurally adjusted ventilatory assist (NAVA) application during non-invasive ventilation (NIV) in Paediatrics | Complete data is not available |
| Zhu2011 | Application of Neurally Adjusted Ventilatory Assist to Children After Congenital Cardiac Surgery | Complete data is not available |
| Emeriaud2019 | Characteristics of the Ventilation Delivered Using Neurally Adjusted Ventilatory Assist in Critically Ill Children | Complete data is not available |
| Lemley2015 | NIV nava improves trigger delay compared to NIV PS in infants with bronchiolitis | Complete data is not available |
| Zhao2013 | Clinical application of NAVA breathing pattern in children after congenital heart disease | The type of study does not match |

**Supplementary Table S3.** Assessment of risk of bias in studies with non-randomized intervention by ROBINS-I

| Study | D1 | D2 | D3 | D4 | D5 | D6 | D7 | Overall |
| --- | --- | --- | --- | --- | --- | --- | --- | --- |
| Xiao2021 | moderate | Low | Low | Low | moderate | no information | moderate | moderate |
| Spinazzola2020 | moderate | Low | Low | Low | moderate | no information | moderate | moderate |
| Ducharme-Crevier2015 | Low | Low | Low | Low | Low | no information | Low | Low |

D1: Bias induced by confounding factors.

D2: Bias related to participant selection.

D3: Bias in intervention classification

D4: Bias stemming from deviations in intended interventions

D5: Bias due to missing data

D6: Bias in outcome measurement

D7: Bias in the selection of reported results

**Supplementary Table S4.** Assessment of risk of bias in Cohort studies by NOS

| Study | D1 | D2 | D3 | Overall |
| --- | --- | --- | --- | --- |
| Piastra2014 | 3 | 1 | 2 | 6 |
| Chidini2021 | 3 | 1 | 3 | 7 |
| Assy2019 | 3 | 0 | 2 | 5 |
| Liu2022 | 4 | 1 | 2 | 7 |

D1: Selection

D2: Comparability

D3: Outcome

**Section: Supplementary Outcomes**

**Supplementary Figure S1: Forest plot demonstrating pooled results for Pmean**

**
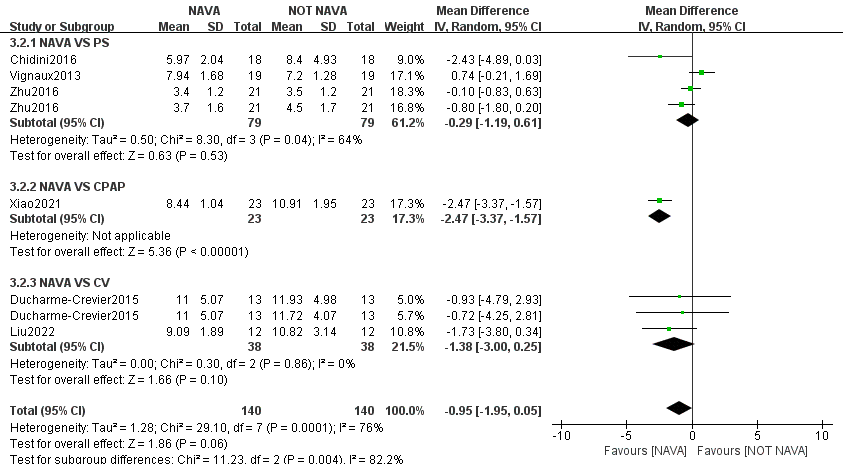
**

**Supplementary Figure S2: Forest plot demonstrating pooled results for PEEP**

**
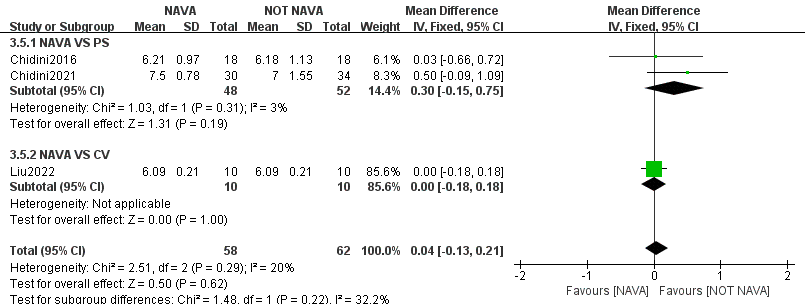
**

**Supplementary Figure S3: Forest plot demonstrating pooled results for TV**

**
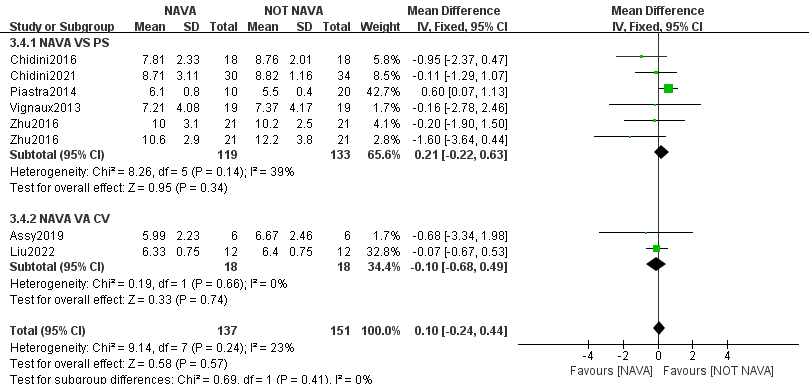
**

**Supplementary Figure S4: Forest plot demonstrating pooled results for FiO_2_**

**
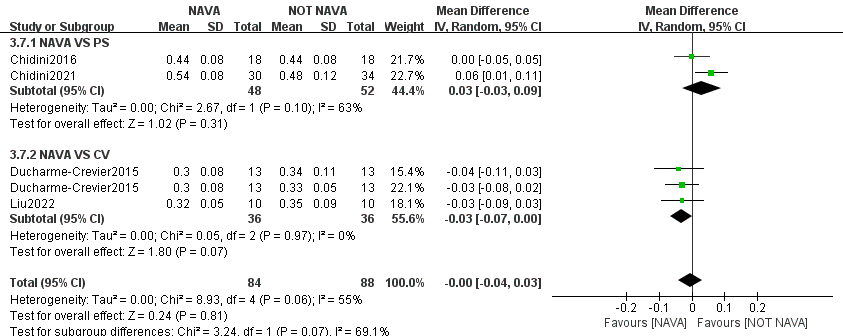
**

**Supplementary Figure S5: Forest plot demonstrating pooled results for pH**


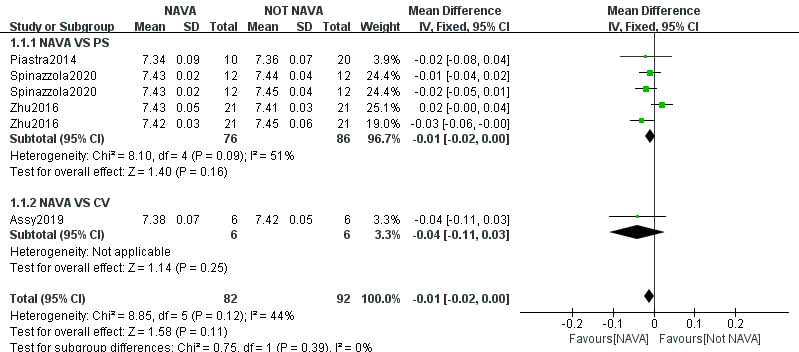


**Supplementary Figure S6: Forest plot demonstrating pooled results for pCO_2_**

**_
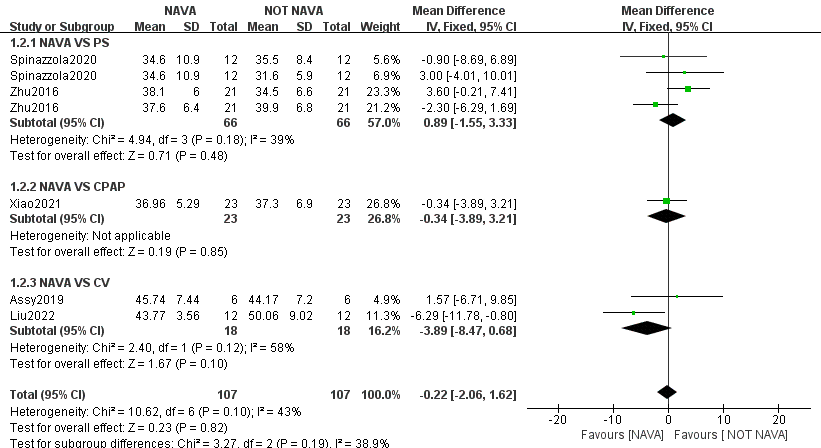
_**

**Supplementary Figure S7: Forest plot demonstrating pooled results for SpO_2_**

**_
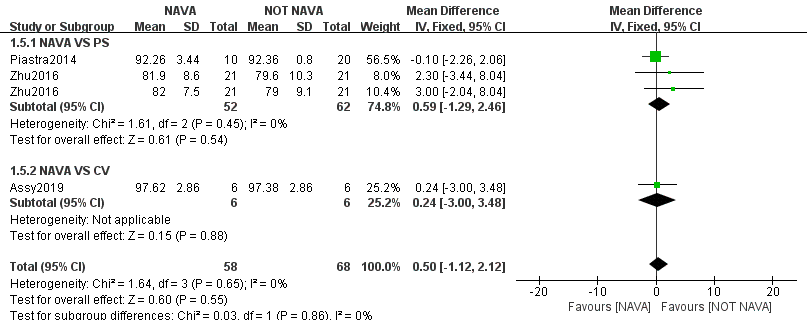
_**

**Supplementary Figure S8: Forest plot demonstrating pooled results for EAdimax**

**
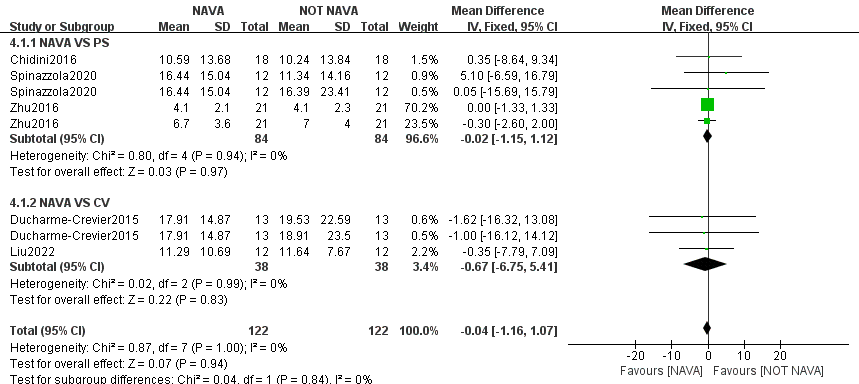
**

**Supplementary Figure S9: Forest plot demonstrating pooled results for EAdimin**


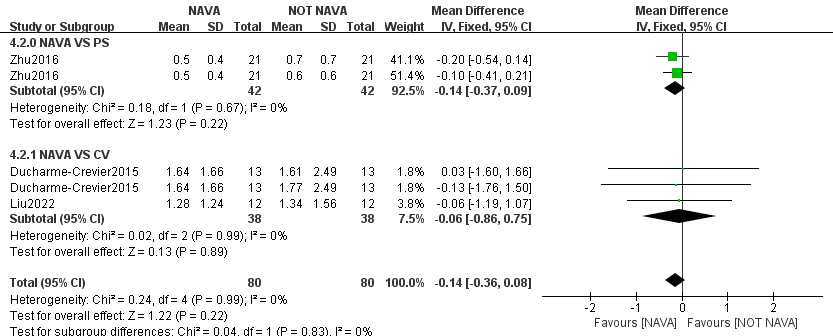

Supplement: Supplementary file 1 [file Table1.docx]
